# Thermal Transient Performance of PEM Fuel Cells in Aerospace Applications: A Numerical Study

**DOI:** 10.1021/acs.energyfuels.4c04834

**Published:** 2025-04-15

**Authors:** Mehdi Seddiq, Mohammad Alnajideen, Rukshan Navaratne

**Affiliations:** † College of Physical Sciences and Engineering, Cardiff University, Cardiff CF24 3AA, U.K.; ‡ City University of London, School of Science and Technology, 280 St John Street, London EC1 V 4PB, U.K.

## Abstract

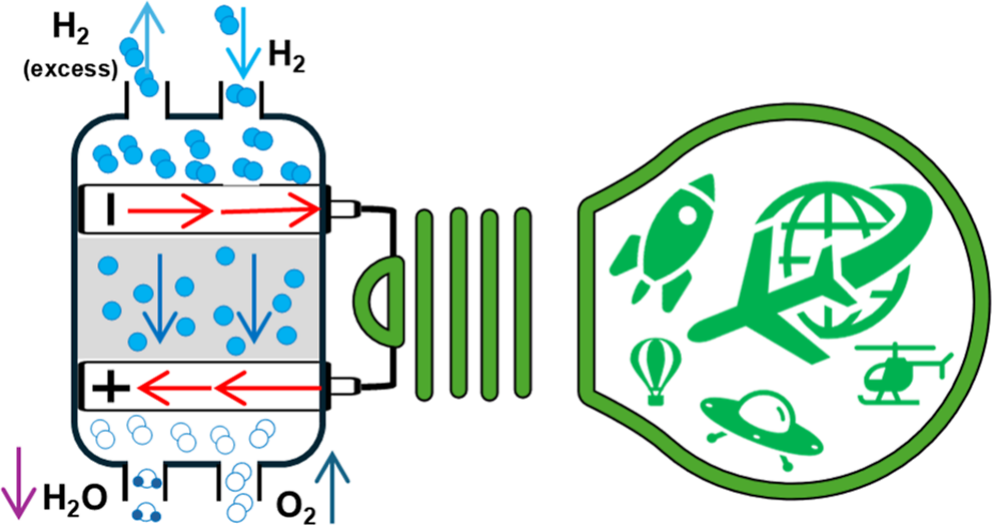

Polymer electrolyte membrane fuel cells (PEMFCs) are gaining attention as viable energy carriers for future aerospace propulsion systems due to their high-power density, lightweight and compact design, zero emissions, scalability, quiet operation, and relatively reliable performance. However, maintaining optimal performance and durability under transient thermal conditions remains a critical challenge, particularly in aerospace environments. Despite extensive research on PEMFCs, the transient thermal effects remain underexplored. This study employs a validated numerical simulation model to investigate the transient responses of a PEMFC subjected to thermal shock cycles, where the bipolar plate walls experience abrupt temperature drops to 10 °C for durations of 3 to 19 s. The simulation model was benchmarked against experimental data from the literature, demonstrating deviations of less than 10% in the polarization curves, confirming its reliability for predicting transient behaviors. Results reveal that during these thermal shocks, the current density decreases by approximately 15%, from 9263 A/m^2^ at 50 °C to 7709 A/m^2^ at 10 °C, with recovery times exceeding 4 s. Significant deviations were observed in oxygen concentration, particularly at the cathode catalyst layer, where minimum levels decreased by over 20%. Similarly, the water content in the membrane showed an overshoot above steady-state levels postrecovery, remaining elevated for extended periods. Liquid water saturation in the gas diffusion layers (GDLs) increased significantly near the hydrogen inlets during cold conditions, obstructing reactant flow and further impacting performance. This study provides detailed predictions of the steady-state and transient responses of PEMFCs to temperature reduction cycles. The findings contribute to advancing thermal management strategies and improving system resilience under transient conditions, thereby addressing a key challenge in sustainable aviation.

## 1 Introduction

Polymer electrolyte membrane fuel cells (PEMFCs) are increasingly considered a promising energy solution for aerospace applications due to their high-power density, lightweight design, and environmentally friendly operation.^1−5^ Unlike conventional fuel technologies, PEMFCs produce only water as a byproduct,^6^ aligning with global initiatives to reduce carbon emissions and enhance sustainability in aviation. Their compact size and scalability enable integration into a variety of aerospace platforms, ranging from unmanned aerial vehicles (UAVs) to commercial aircraft.^7^ Moreover, PEMFCs operate quietly and efficiently under varying environmental conditions, making them suitable for both commercial and military applications where noise reduction and stealth are critical.^8−11^

Despite their advantages, widespread adoption of PEMFCs in aerospace remains challenging due to high production costs, primarily associated with platinum catalysts, and durability concerns linked to membrane and catalyst degradation.^12−16^ This degradation can occur through chemical, mechanical, and thermal pathways, leading to a reduction in performance and lifespan. Effective water and thermal management are also essential to ensure optimal performance. Excess water accumulation can block reactant gases, whereas inadequate hydration can reduce membrane conductivity, impacting fuel cell efficiency.^17,18^

Unlike automotive PEMFCs, which are optimized for rapid start-up and frequent load variations,^19^ aerospace PEMFCs must function reliably under extreme altitudes, variable temperatures, and reduced atmospheric pressure. This necessitates robust design strategies that prioritize durability and fuel efficiency over cost, given the premium placed on reliability and safety in aerospace missions.^20^ The design must accommodate stringent weight and volume constraints while maintaining stable performance in low-pressure environments^11^^,^.^21^ In contrast, automotive PEMFCs focus on optimizing volumetric and gravimetric power density to reduce system weight and enhance vehicle range, as well as minimizing costs to ensure market competitiveness. Advances in catalyst development, such as platinum group metal-free (PGM-free) catalysts, and improvements in membrane durability under varying operating conditions, are crucial to achieving these goals^16^^,^^19^^,^.^22^

Automotive PEMFCs typically operate within stable environments but must handle frequent load variations due to acceleration and deceleration cycles. Efficient waster and heat management is crucial to prevent membrane flooding or drying, particularly under subfreezing or high-humidity conditions.^23,24^ Thermal management systems maintain optimal operating temperatures to ensure consistent performance. Aerospace PEMFCs, however, face unique environmental challenges, including reduced oxygen availability at high altitudes and extreme temperature variations.^25^ Lower oxygen partial pressure necessitates advanced air compression systems or oxygen-enrichment systems to maintain adequate reaction kinetics.^26^ Aerospace PEMFCs often incorporate specialized materials and coatings to withstand radiation or corrosive atmospheric conditions.^27,28^

Durability remains a concern for PEMFCs applications in automotive and aerospace sectors. Automotive PEMFCs have lifespans of approximately 5000 to 8000 h, matching typical vehicle life expectancy.^29−31^ Their design focuses on mitigating degradation, membrane thinning, and gas diffusion layer wear often caused by dynamic load cycling and frequent start–stop conditions.^32,33^ In contrast, aerospace PEMFCs require significantly longer operational lifespans with minimal maintenance, particularly for space missions or UAVs, where repairs are infeasible.^34^ These systems are designed to endure continuous operation over tens of thousands of hours, necessitating superior material stability and innovative strategies to mitigate mechanical and chemical degradation.^35^ Several studies have discussed differences and challenges between automotive and aerospace PEMFC designs.^24,28,34,36^

PEMFCs generally operate at relatively low temperatures, which limit their performance and efficiency in certain applications.^37^ Increasing operating temperatures can enhance performance but presents material and engineering challenges, such as maintaining membrane hydration and stability.^37^ In addition, hydrogen storage and distribution logistics further complicate large-scale PEMFC deployment,^38,39^ and safety concerns due to hydrogen’s flammability necessitate rigorous management.^39,40^

Advances in materials science and engineering are enabling more efficient and cost-effective PEMFCs. Innovations in catalysts,^41,42^ membrane durability,^42,43^ and system integration^42^^,^^44^ are steadily reducing costs and improving the viability of this technology. A summary of the key challenges (or barriers) is presented in Figure S1 (appendix A), identifying essential research and development prioritize for improving PEMFC performance and ensuring material integrity before commercial deployment.

In a single PEMFC (Figure S2,^45^ appendix A), electricity generation occurs via reaction between oxygen and hydrogen, separated by a porous membrane with a thin catalyst layer (CL). At the anode (fuel side), hydrogen splits into protons and electrons; protons migrate through the membrane to the cathode, reacting with oxygen to produce water. Electrons flow through conductive bipolar plates (BP), generating electrical current for external circuits. Channels within bipolar plates distribute hydrogen and oxygen, while the gas diffusion layer (GDL) between the plates and membrane ensures even gas distribution, manages excess water, and conducts electricity. Individual PEMFCs generate approximately 0.5–0.8 V each,^46^ requiring stacking of multiple cells to increase voltage and power. The combined structure (membrane, catalyst layers, and GDL) forms the Membrane Electrolyte Assembly 11 11. Auxiliary systems, including water and thermal management systems, support PEMFC operation. Proper humidity control prevents membrane dehydration or water blockage in gas channels.^47^ Thermal management systems regulate temperature, maintaining the optimal performance range.^48^ Understanding fuel cell performance under steady-state and transient conditions requires experimental and computational investigations.

Zhang et al.^49^ experimentally studies the dynamic behavior of high-temperature PEMFCs under load variations, identifying hysteresis in polarization curves, particularly evident at lower currents and higher voltages. Saleh et al.^50^ experimentally validated a mathematical model for a self-humidifying PEMFCs, focusing on voltage and temperature changes under varying loads. While some researchers adopt semiempirical models for simplicity and parameter fitting,^51−53^ such models typically offer limited insights into internal fuel cell processes. For instance, Akimoto and Okajima^54^ employed a semiempirical approach to assess the impact of temperature on voltage–current density relationships.

Dynamic models address transient fuel cell behavior in response to changing loads or operating conditions,^55^ often simplifying the computational complexity by using reduced-dimensional approaches. Pathapati et a.^56^ proposed a dynamic model to study variations in cell voltage, temperature, pressure, and inlet flow rates in response to sudden load changes. Shamardina et al.^57^ developed a dynamic model to evaluate electrochemical impedance under step changes in potential, current and current interruptions.

Rabbani and Rokni^58^ studied transient nitrogen accumulation in PEMFC anodes using commercial software tools, exploring purging strategies to enhance performance. Lan and Strunz^59^ used equivalent electric circuit modeling to study auxiliary systems during transient operations, whereas Zou and Kim^60^ employed MATLAB- Simulink for fuzzy controller-based thermal management. Similarly, Ceylan and Devrim^61^ developed a MATLAB-Simulink dynamic model incorporating fuel cell systems with solar energy integration, batteries, electrolyzers, and economic analysis.

Comprehensive understanding of electricity generation in PEMFCs requires detailed modeling of fluid dynamics and concentration distribution through full transport equations, despite high computational demands. Yan et al.^62^ implemented a three-dimensional model to study transient current density and mass transport in serpentine-layout channel designs. Goshtasbi et al.^63^ investigated transient responses to voltage and current variations, highlighting the critical role of water transport and microstructural features. Bodner et al.^64^ examined hydrogen starvation effects during start-up via ANSYS-Fluent, proposing a scheme for larger time steps in computations. Wang et al.^65^ presented a two-dimensional model to investigate the transient effects of current change on parameters such as temperature and humidity at cathode catalyst layer, membrane water content, and output voltage, exploring how different channel widths impact results. Kravos et al.^66^ presented a two-phase transient model, addressing crucial transport phenomena within segmented membrane electrode assemblies.

Temperature significantly influences PEMFC performance, as effective heat management is essential for optimized operation. Elevated temperatures enhance reaction kinetics but may increase voltage losses.^67,68^ Operating at higher temperatures reduces liquid water blockage through enhanced vaporization, leveraging generated heat.^69^ Conversely, lower temperatures simplify water management and accelerate start-ups, while reducing corrosion and thermomechanical stress, extending lifespan.^70^ Therefore, understanding temperature effects on membrane conductivity and water transport diffusivity is vital. Yan et al.^71^ experimentally analyzed PEMFC cold start-up behavior at different temperatures, assessing irreversible performance degradation at subfreezing temperatures. Adzakpa et al.^72^ developed a three-dimensional dynamic model addressing steady-state temperature nonuniformities and transient responses in air-cooled fuel cells. Ondrejička et al.^73^ numerically investigated steady-state temperature impacts on voltage losses, identifying optimal operating conditions across different voltage ranges and transient power fluctuations.

Current research on PEMFCs has primarily focused on steady-state performance, leaving transient thermal behavior underexplored. Understanding how PEMFCs respond to sudden temperature changes is crucial for improving system resilience and reliability in aerospace applications. This study addresses this gap by investigating the transient thermal performance of PEMFCs under temperature drop cycles. Using a validated numerical model, we analyze the effects of abrupt temperature reductions on key performance parameters, including current density, reactant distribution, and water content. The insights gained from this study contribute to optimizing thermal management strategies and enhancing PEMFC durability in real-world aerospace scenarios.

## 2 Methodology

### 2.1 PEMFC: Simulation Setup

This study employs a PEMFC consisting of a parallel layout with a counter-flow configuration (Figure S3, Appendix). The choice of this design is guided by its advantages under steady-state, moderate-load conditions. Parallel channel designs provide lower pressure drops than serpentine or interdigitated flow fields, thus reducing auxiliary power losses and enhancing system efficiency, which is vital for energy-limited aerospace applications.^74−77^ Counterflow arrangements further improve thermal and reactant distribution, reducing temperature gradients and mitigating potential hotspots or uneven water distribution, thus stabilizing performance under moderate current densities.^78−82^ The result is a more stable cell performance under low to moderate current densities, as observed in previous studies.^78,81,83−85^ Although parallel-counterflow configurations might not outperform serpentine channels at high loads or transient conditions, their strengths align closely with aerospace application priorities: lightweight systems, minimal energy losses, and operational stability.^86−89^ As noted in previous work,^78,79,81,83−85,90−92^ the parallel-counterflow combination offers a promising trade-off between efficiency, simplicity, and reliability, which are essential for aerospace systems constrained by weight and operational stability.

Table 1 provides cell specifications, and Table 2 illustrates simulation conditions for the flows at the cell channels’ inlets. Uniform mass fluxes with specified mole fractions have been considered at the inlets. ANSYS 2021-R1 software was employed to simulate the chemical reaction and calculate species concentration variations, fluid velocity, pressure, temperature changes and electricity generation within a single cell. The meshing and numerical solution procedure for cell simulations is available in the Appendix.

**Table 1:** Specifications of the PEMFC Used in This Study

parameter	value	unit
cell planar dimensions	30.00 × 30.00	mm × mm
channel height	1.00	mm
channel width	1.00	mm
bipolar plate (BP) maximum thickness	1.50	mm
GDL thickness	300.00	μm
CL thickness	12.90	μm
membrane thickness	108.00	μm
GDL porosity	0.60	
CL porosity	0.20	
CL absolute permeability	2.00 × 10^–13^	m^2^
GDL absolute permeability	3.00 × 10^–12^	m^2^
anode reference current	10000.00	A/m^2^
cathode reference current	10.00	A/m^2^
anode/cathode activation energy	8314.34	J/mol
temperature for reference current	70.00	°C
steady-state voltage	1.23	V
BP material	Carbon graphite	

**Table 2:** Flow Conditions at the Channel Inlets for all Simulations in This Study

parameter	value	unit
pressure (both sides)	1.00	bar
inlet temperature (both sides)	50.00	°C
inlet relative humidity for anode side	100.00	%
inlet relative humidity for cathode side	50.00	%

The chosen cell planar dimensions (30.00 × 30.00 mm) represent a simplified geometry suitable for numerical modeling and detailed computational analyses. While this smaller scale may limit the direct representation of thermal gradients found in practical, full-scale PEMFC stacks used in aerospace applications, it remains effective for investigating the fundamental transient thermal responses and associated performance dynamics. A comprehensive evaluation of larger-scale cells or stack-level modeling would be beneficial for further validation and for capturing the effects of realistic temperature distributions in actual operational environments.

### 2.2 PEMFC: Computational Configuration

Inlet mass flow rates include vapor and dry reactant mass flow, calculated using stoichiometric ratios (anode: 1.6, cathode: 2.5 at a reference current density of 10,000 A/m^2^). Actual stoichiometric ratios increase as output current densities decrease. Constant temperature boundary conditions (50 and 10 °C) are applied to the bipolar plate (BP) walls, including the back terminal surfaces, side walls perpendicular to the membrane, and walls around the channels. These walls are assumed to be controlled by the thermal management system. Conjugate conditions are applied at the BP-GDL interface. A pressure outlet boundary condition is used for the outlets. Except for BP walls, all other side boundaries surrounding the membrane electrode assembly 11 are considered walls with zero fluxes for pressure, temperature, species, and electric potential. The cell terminal voltage is kept constant at 0.55 V, a typical operating voltage for fuel cells close to the maximum power point. High current densities at this voltage result in liquid water formation in parts of the GDL and CLs. Current density, an output quantity, is calculated by integrating over the back surface of the bipolar plates, and average values from the anode and cathode are reported.

To study transient and steady-state effects of temperature changes, a series of numerical experiments with different temperature cycles are conducted. Normally, the BP wall temperature is the same as the inlet flow temperature (50 °C). A thermal shock is applied by reducing the BP external walls temperature to 10 °C, creating spatial temperature gradients within the stack during transient conditions. The temperature then returns to the original value, completing a thermal cycle. The temperature is represented by *T*_CL,c_ and *T*_CL,a_, referring to the average of maximum and minimum temperatures within the cathode and anode catalyst layers, respectively. In this study, the bipolar plates are modeled with constant temperature conditions of 50 and 10 °C to reflect their high thermal conductivity of graphite, which minimizes temperature differences across the plates. These specific temperatures represent typical operating and extreme cold-start conditions relevant to aerospace applications. This simplification enables the study to isolate and analyze the transient thermal effects at the interface between the bipolar plates and other components without introducing additional complexity from internal temperature gradients within the plates.

### 2.3 PEMFC Model

Fluent software, along with the PEMFC module, is used to compute the two-phase multispecies fluid flow, heat transfer, and charge transport in a three-dimensional domain. The flow is considered laminar (Reynolds number is approximately 418) and incompressible, and the equations for liquid water transport are included for both porous parts and channels. The basic equations^93,94^ used for the conservation of mass and momentum are as follows
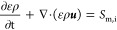
1
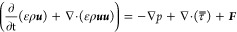
2whereas ρ, ***u***, ε,  and ***F*** represent the density, velocity vector, porosity, stress tensor, and external body forces, respectively. *S*_m,i_ denotes the rate of mass density change for the species i due to its consumption or production. This value is negative for hydrogen and oxygen in anode and cathode catalysts, positive for water in cathode catalyst, and zero otherwise.

Equations 1 and 2 are not solved for the bipolar plates (BPs), which are solid structures, nor for the membrane. In the membrane and catalyst layers (CLs), water exists in the form of a dissolved phase, and its transport in these regions is calculated using alternative equations based on water content. The distribution of species is calculated by solving separate transport equations for each species *i*, as given by Equation (3)
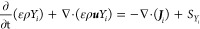
3where *Y*_i_ is the local mass fraction for the species i, ***J***_i_ is a vector denoting the diffusive mass flux for the species i, and *S*_Yi_ is the source term. The diffusion term **J**_i_ is governed by gradients of concentration and temperature.

The Full Multicomponent Diffusion method, which accounts for the species binary mass diffusion coefficients and thermal diffusion coefficients, has been used to enhance the accuracy of the diffusion flux calculation. The source term *S*_*Yi*_ includes the production/consumption terms caused by chemical reaction and a term for addition from the dispersed phase. The species transport equation is solved for all species except for nitrogen, which is calculated by a simpler equation (eq 4). The nitrogen concentration is typically determined using an assumption of its inert behavior, simplifying the model.
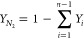
4where *n* = 4, is the number of species.

The transport equations for liquid water in the gas diffusion layers (GDL) and catalyst layers (CLs), and membrane are described based on liquid saturation, *s* which is defined as the fraction of the pore space filled by the liquid water. The liquid saturation *s* is calculated using a transport equation as follows
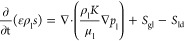
5where; ρ_l_ and μ_l_ are the density and dynamic viscosity of the liquid water, respectively. *K* is the effective permeability which is the production of the absolute and relative permeabilities, *S*_gl_ is the rate of mass exchange between gas and liquid phases, and *S*_ld_ is the rate of mass exchange between liquid and dissolved phases.

The calculation of liquid water transport in gas channels provides more accurate results by predicting the pressure drop increase due to the presence of liquid water
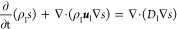
6where **u**_l_ is the velocity of the liquid water in channels, and it is a fraction of the local velocity of the gas. D_l_ is the liquid water diffusion coefficient in the channels.

The energy equation for the single-phase regime is given by
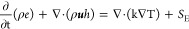
7where *e* and *h* are the internal energy and enthalpy, *T* is the temperature, *k* is the thermal conductivity and *S*_E_ is the thermal source term which includes heat generation caused by the chemical reaction and ohmic losses.

The electric potential is calculated based on transport equations for the solid and membrane phases separately
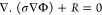
8where Φ is the electric potential, σ is the electric conductivity, and *R* is the volumetric transfer current. *R* is zero all over the domain except for the CLs. It depends on the molar concentrations of the reactants and temperature and is calculated based on a general formulation of the Butler–Volmer function
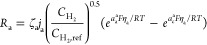
9
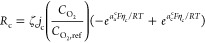
10where indexes *a* and *c* denote anode and cathode sides respectively, ζ is specific active surface area, *j* is the reference exchange current density explained below, *C* is the local concentration for a reactant (oxygen in cathode and hydrogen in anode), index ref denotes the reference concentration, *a*^a^ and *a*^c^ are the transfer coefficients of anode and cathode respectively (each anode and cathode has its set of transfer coefficients), *F* = 96485 C/mol is the Faraday constant and η is the surface overpotential. The reference exchange current density is a function of the temperature
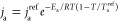
11
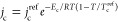
12where *j*^ref^ is the value at the reference temperature *T*^ref^, and *E* is the electric potential.

Further details about the governing equations can be found in ANSYS-Fluent 2021 R1 Theory Guide.^95^

## 3 Results and Discussions

### 3.1 Validation of the Simulation Model

To validate the simulation model developed for this study, a mesh independence study was conducted to select an appropriate mesh size with a reasonable number of computational elements. For model validation, the experimental study of Najmi et al.^96^ with a parallel channel were used as benchmarks. The resulting voltage–current density relationships, known as the polarization curves, are shown and compared against the experimental data from the benchmark in Figure S4 (appendix). Compared to Najmi et al.,^96^ the model shows agreement with a deviation of less than 10% in the range of 0.35–0.85 V. These deviations are considered acceptable, indicating that the results of the present model are in particularly good agreement with the benchmark analysis. While the current model validation relies on steady-state experimental data due to limited availability of transient benchmarks, future studies will incorporate transient experimental validation to further confirm the accuracy of the transient predictions.

### 3.2 Single Thermal Shock

In the first simulation, while the fuel cell operation is in a fully steady state, at *t* = 2*s*, the BP walls are dropped to 10 °C, and they remain at this temperature for 19 s. The temperature then returns to normal. Another simulation is also conducted with a shorter period of 3s for the cold temperature to study the effect of the cycle duration. The variations in the average maximum and minimum temperatures within the cathode catalyst layer, *T*_CL,*c*_ are shown in Figure S5 (appendix). After the temperature drops at the BPs walls, CL is affected by more than 6 °C reduction within 10 ms. In about 1*s*, the average temperature is reduced by approximately 37.5 °C and to the end of the cold conditions at *t* = 21 s, it undergoes a further reduction of only 0.2 °C.

**Figure 1:**
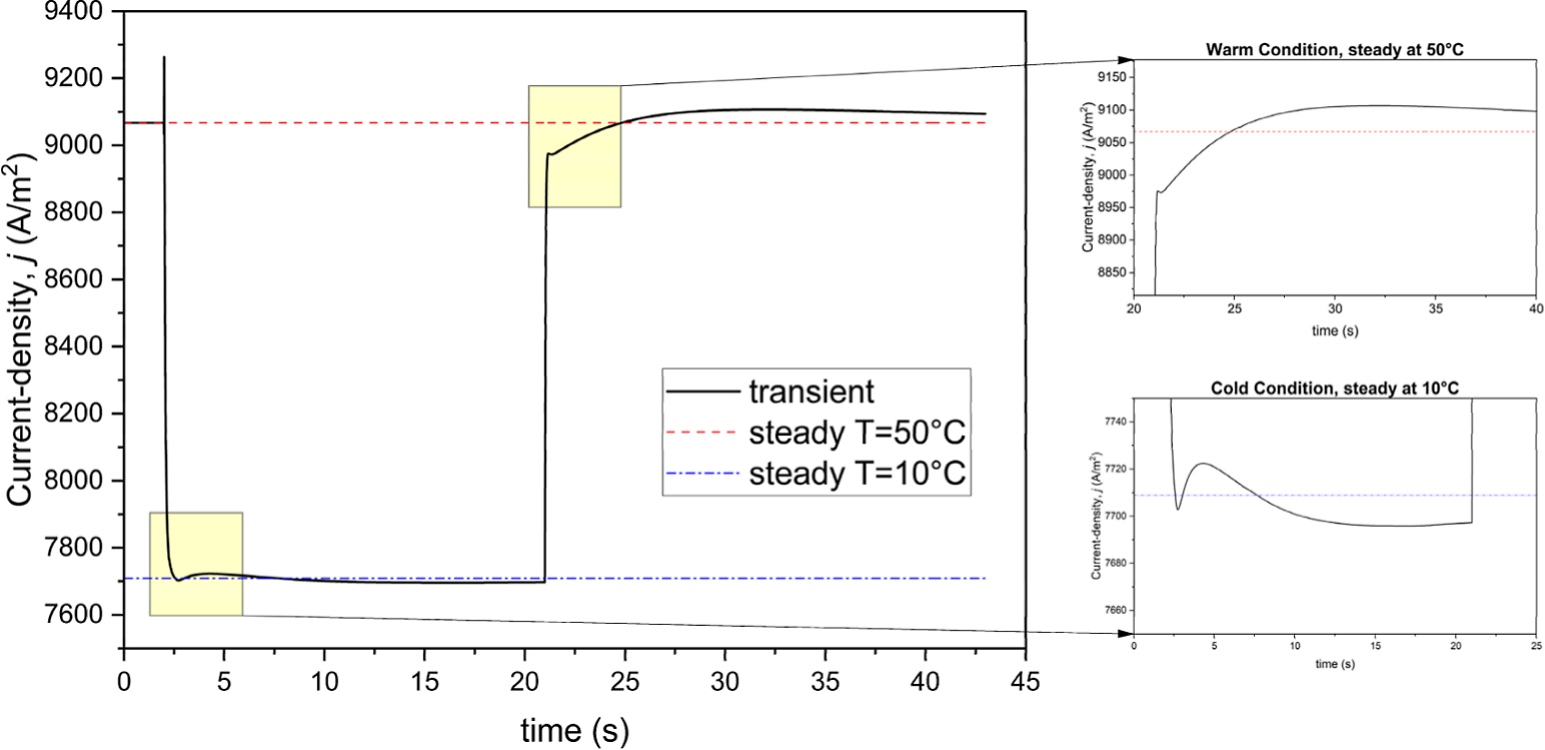
Variations in current density during the transient simulation with a single thermal cycle (inset figures show detailed views for specific time intervals at cold and warm steady-state conditions).

Figure 1 illustrates the current density, *j*, of the simulated fuel cell. Under steady-state conditions at 10 °C, the current density is 7709 A/m^2^, which is significantly lower than the 9263 A/m^2^ observed at 50 °C. This reduction indicates that the fuel cell delivers ∼15% less power during the colder part of the cycle. During cold conditions, the current density stabilizes slightly above the steady-state value. Afterward, the fuel cell underperforms for 3.8 s, with the current density 1% below the normal steady-state value. A shorter cold cycle (see Figure S6) reduces the intensity of underperformance but extends its duration to 4.8 s. By *t* = 31 s, the current density remains 0.3% above the steady-state value.

**Figure 2:**
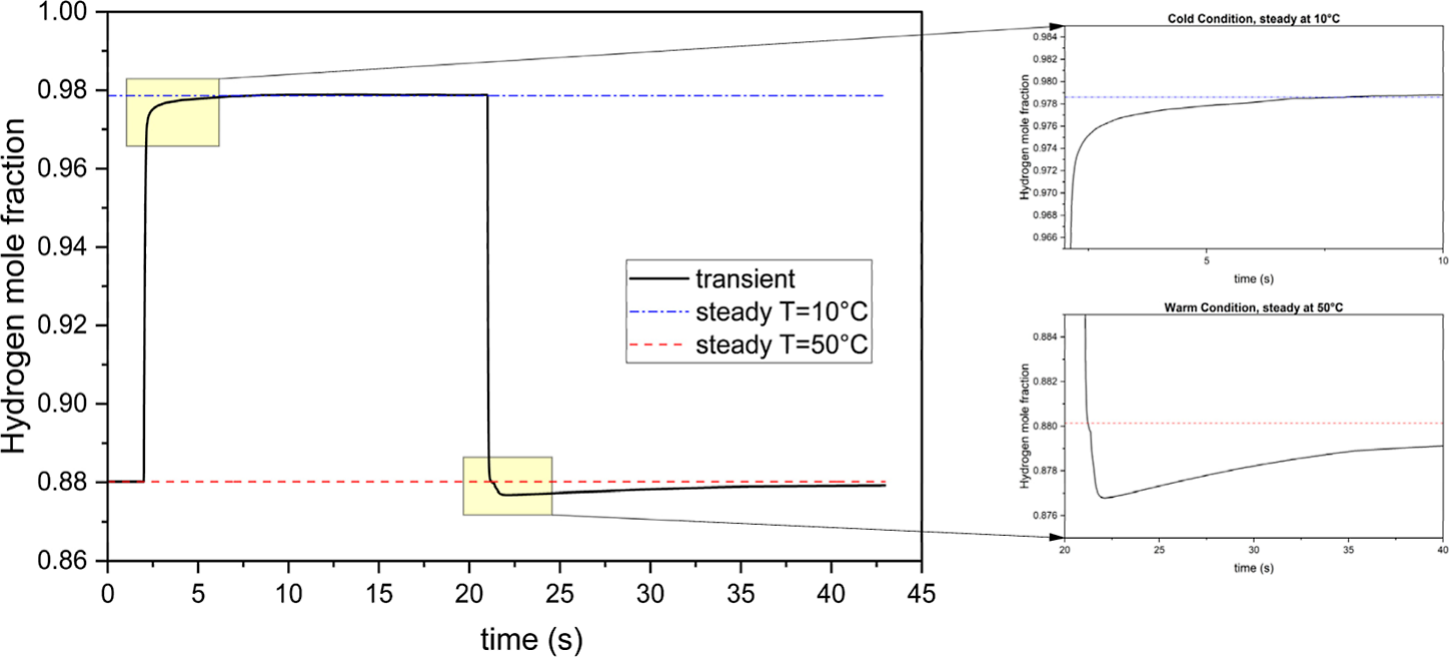
Minimum mole fraction of hydrogen at anode catalyst layer-gas diffusion layer during the transient simulation with a longer-period thermal cycle (inset figures show detailed views for specific time intervals at cold and warm steady-state conditions).

As the simulation began, the fuel cell operated steadily until the temperature of the BP walls was abruptly reduced to 10 °C. This sudden drop in temperature triggered a series of changes within the cell. Notably, the minimum hydrogen mole fraction *f*_*H*_2__, at the anode catalyst layer-gas diffusion layer (CL-GDL) interface increased significantly. This increase remained stable until the temperature returned to normal. However, the return to normal conditions initiated a rapid reduction in *f*_*H*_2__, pushing it below the steady-state levels. Gradually, over time, *f*_*H*_2__ began to approach its steady-state value once again. This dynamic behavior had a profound impact on the current density within the cell. As can be seen in Figure 2, the minimum hydrogen mole fraction at the anode CL-GDL interface clearly demonstrates these fluctuations. The data suggests that the initial low current density following the thermal shock correlates with the observed decline in *f*_H_2__ values below the steady-state levels. This correlation highlights the sensitivity of the fuel cell’s performance to changes in the hydrogen mole fraction at critical interfaces.

During cold conditions, the increase in the minimum hydrogen mole fraction is primarily due to the lower current generation and reduced hydrogen consumption rates. In addition, since the anode inlet is fully humidified, the lower temperatures cause more water to saturate, leaving less water vapor in the pores. This results in a higher mole fraction of hydrogen. To understand the causes of the reduction in hydrogen mole fraction after the cold temperature period, we can look at Figure S7 (appendix), which shows the liquid water saturation (*s*) contours in the anode’s CL-GDL at *t* = 21 s. This moment is shortly before the temperature returns to normal, and the fuel cell’s performance is steady at 10 °C. Before the temperature drops, the maximum water saturation in the anode occurs near the edge, close to the inlet, with no significant variations in saturation elsewhere. However, when the BP’ walls and channel walls are cold, and the inlet flow is fully wet at 50 °C, water condenses along the channel, leading to considerable saturation around the inlet. This increase in condensed water within the anode’s porous media, especially near the inlet, remains long after the temperature normalizes. Liquid water obstructs parts of the hydrogen passage near the inlet, where fresh hydrogen is supplied. As a result, hydrogen concentrations decrease downstream, contributing to the observed reduction in hydrogen mole fraction.

**Figure 3:**
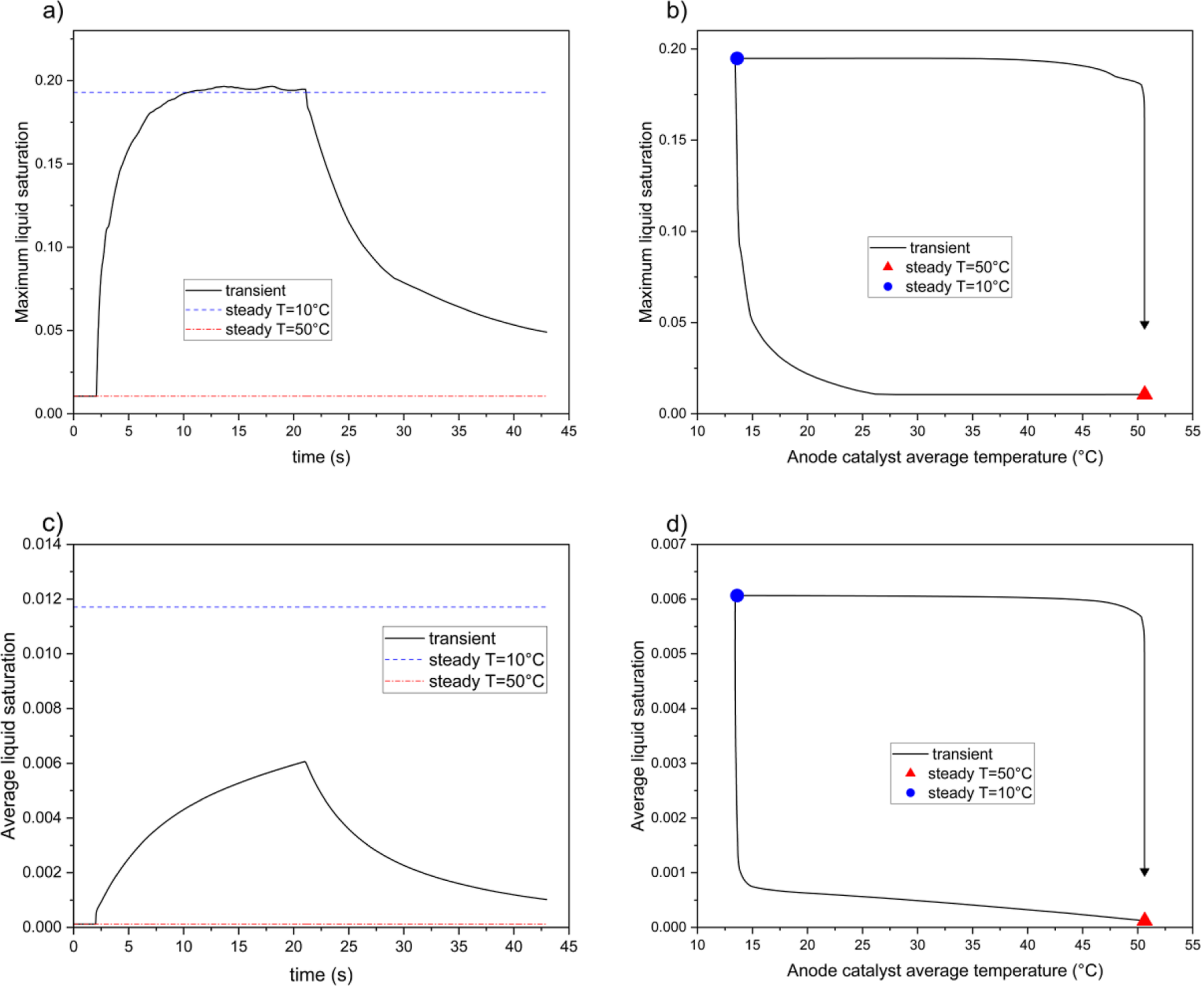
Variations of liquid water saturation inside the anode GDL during a long-period thermal cycle; (a,b) are the maximum liquid saturation over time and with temperature changes, respectively; (c,d) are the average liquid saturation over time and with temperature changes, respectively.

Figure 3 shows the maximum and average values of liquid water saturation (*s*) inside the anode GDL during the simulation. The thermal cycle causes a significant increase in the amount of saturated water within the anode. The maximum saturation is notably higher than the average saturation and exhibits a quicker rise toward the steady-state level (see Figure 3a,c). By the end of the cold conditions, the maximum saturation reaches the steady-state value, despite the average saturation lagging behind. Both curves respond immediately to the thermal shock and start to decline once the temperature returns to normal. However, while the maximum saturation grows rapidly in response to the temperature reduction, the reduction rates of both maximum and average saturations are slow when the temperature normalizes. The anode average temperature curves indicate a hysteresis between saturation values as the temperature varies. Although the saturation threshold is linked to temperature, the saturation changes only slightly during temperature fluctuations. This phenomenon can be explained by two key factors. First, the latent heat of condensation and evaporation for water is high, meaning that even a small change in the amount of saturated water requires considerable heat absorption or rejection. Consequently, condensation and evaporation occur on a time scale longer than temperature changes. Second, in this simulation, the current density follows the temperature variations. As the temperature decreases, less current is generated, resulting in lower water production.

The water saturation distributions in the cathode also provide valuable insights. Figure S8 (appendix) shows the contours for two instances: (a) before the temperature reduction, where conditions are steady at 50 °C, and (b) near the end of the low-temperature period, where conditions are close to steady state at 10 °C. At the normal temperature of 50 °C, saturation is lowest near the inlet because the inlet flow has only 50% relative humidity, and water produced by the reaction is added to the flow as it moves downstream. Liquid water tends to form mainly adjacent to the edges where only one branch of the channel is nearby to carry the excess water. There is also a considerable amount of liquid water close to the outlets where the water generated upstream accumulates. When the temperature is reduced, the relative humidity at the inlet increases, leading to regions with higher water saturation close to the inlet. The areas with low saturation values shift from the inlet toward the downstream sections of the interior branches, close to the downstream collector channel (the vertical channel on the left, as shown in Figure S3 in the Appendix).

**Figure 4:**
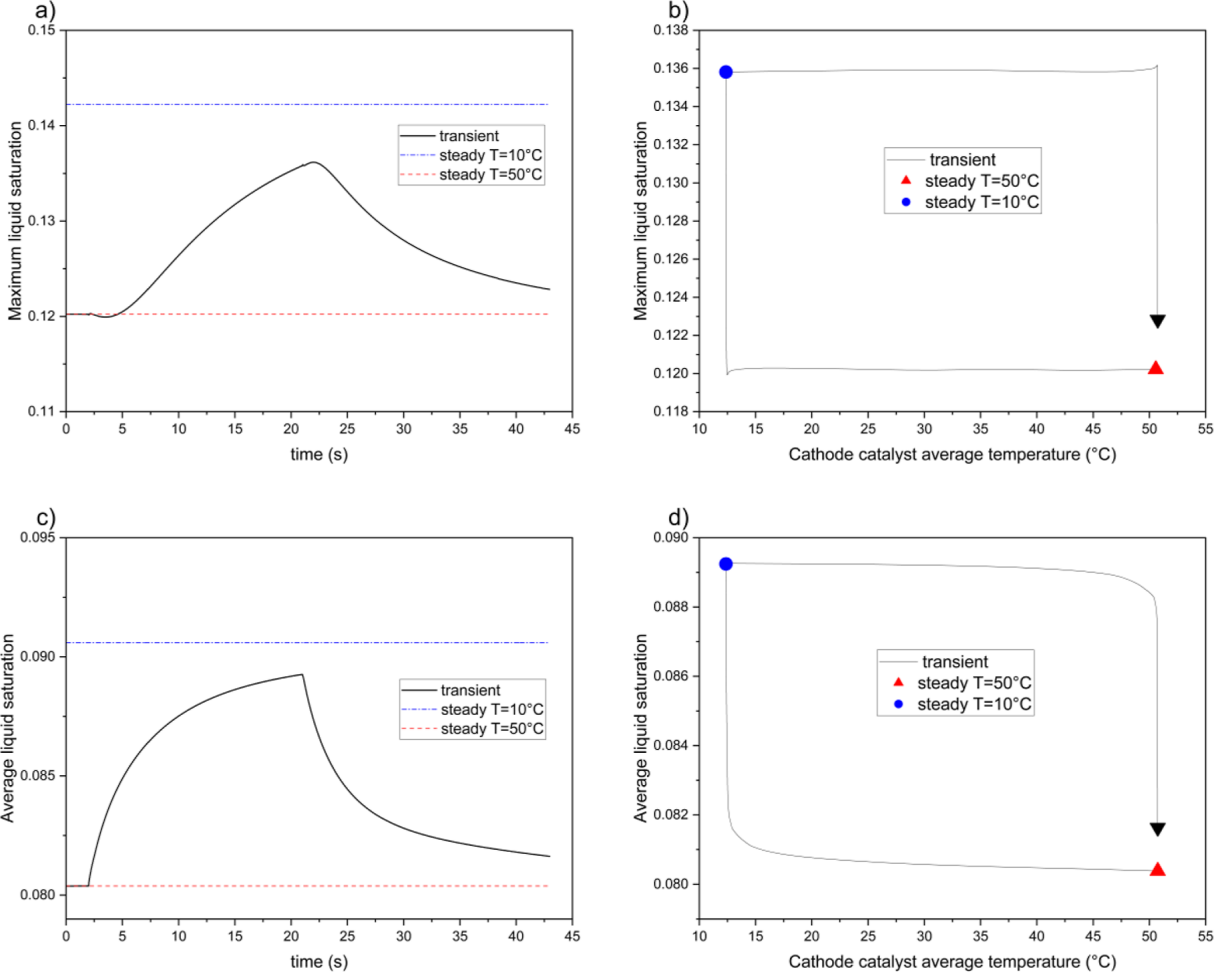
Variations of liquid water saturation inside the cathode GDL during a long-period thermal cycle; (a,b) are the maximum liquid saturation over time and with temperature changes, respectively; (c,d) are the average liquid saturation over time and with temperature changes, respectively.

Figure 4 presents the temporal curves for liquid water saturation inside the cathode GDL. The patterns and amounts of saturation differ between the cathode and anode sides due to water production in the cathode catalyst layer (CL) and less humidity being introduced at the inlets. At the beginning of the cold conditions, the curve for maximum saturation in the cathode decreases but soon starts to increase. Interestingly, it continues to rise for about 1 s after the cold conditions end, although it remains substantially lower than the steady-state value. This behavior is similar to what is observed in Figure 3a for the maximum water content in the membrane, suggesting a strong association between the maximum water content in the membrane and the maximum liquid water on the cathode side. The curve for average saturation, on the other hand, shows a faster response to temperature changes. As the temperature drops, the average saturation increases rapidly. Immediately after conditions return to normal, the average saturation begins to decline.

Figure S9 illustrates the temporal variations of the minimum oxygen mole fraction, *f*_O_2__ at the cathode CL-GDL interface. Unlike the minimum hydrogen mole fraction, which shows a different pattern during cold conditions, the minimum *f*_O_2__ is considerably reduced. This is in stark contrast to the behavior seen in Figure 2 for the minimum hydrogen mole fraction. In fact, the maximum and average *f*_H_2__ in the anode and the maximum and average *f*_O2_ in the cathode (see Figure S10 in the appendix) follow similar patterns to that of the minimum hydrogen. Figure S9 (appendix) also indicates that the minimum *f*_O_2__ at the cathode CL during the cold part of the transient cycle is significantly lower than its steady-state value under cold conditions. This suggests that the phenomenon is even more pronounced if the temperature is rapidly reduced. During most of the cold conditions, the minimum *f*_O_2__ exhibits an increasing trend, partially compensating for the dramatic initial drop after the temperature reduction. However, the rate of increase is low, and by the end of the cold conditions, the curve does not reach the steady-state value. This pattern is like the case with a shorter period of temperature reduction.

It is noteworthy that oxygen concentrations and current density are mutually interconnected. According to the reaction kinetics in eq 10, the rate of the chemical reaction in the cathode is proportional to the oxygen concentration, meaning higher *f*_O_2__ results in increased current density. However, an increase in current density leads to more oxygen consumption, which tends to reduce *f*_O_2__.

The distributions of oxygen and nitrogen concentrations at a time close to the end of the cold conditions are presented in Figure S11 (appendix). Oxygen concentrations are lower in two regions: first, adjacent to the edges where only one branch of the channel supplies oxygen, and second, in a central-left area near the downstream collector channel (the vertical channel on the left, as shown in Figure S3), where the oxygen supply flow is weaker. Contours at *t* = 1.5 s during the steady-state condition at 50 °C (see Figure S12) indicate that while temperature variations change the mole fraction amounts, the locations of high and low concentrations remain almost unchanged. The oxygen mole fraction values near the edges are lower at both cold and normal temperatures but undergo further reductions during the cold cycle.

Several factors can potentially cause reductions in oxygen concentration. Comparing the oxygen and nitrogen concentrations in Figure S11 with the water saturation contours *t* = 20.5 s in Figure S8 provides valuable insights. The oxygen mole fraction is inversely correlated with the nitrogen mole fraction, indicating no significant blockage inhibiting gas flow, including oxygen. In addition, there is no substantial water production in the low-oxygen areas, suggesting no excess oxygen consumption in these regions. Overall, the low-oxygen regions are formed where the oxygen supply does not match its consumption.

**Figure 5:**
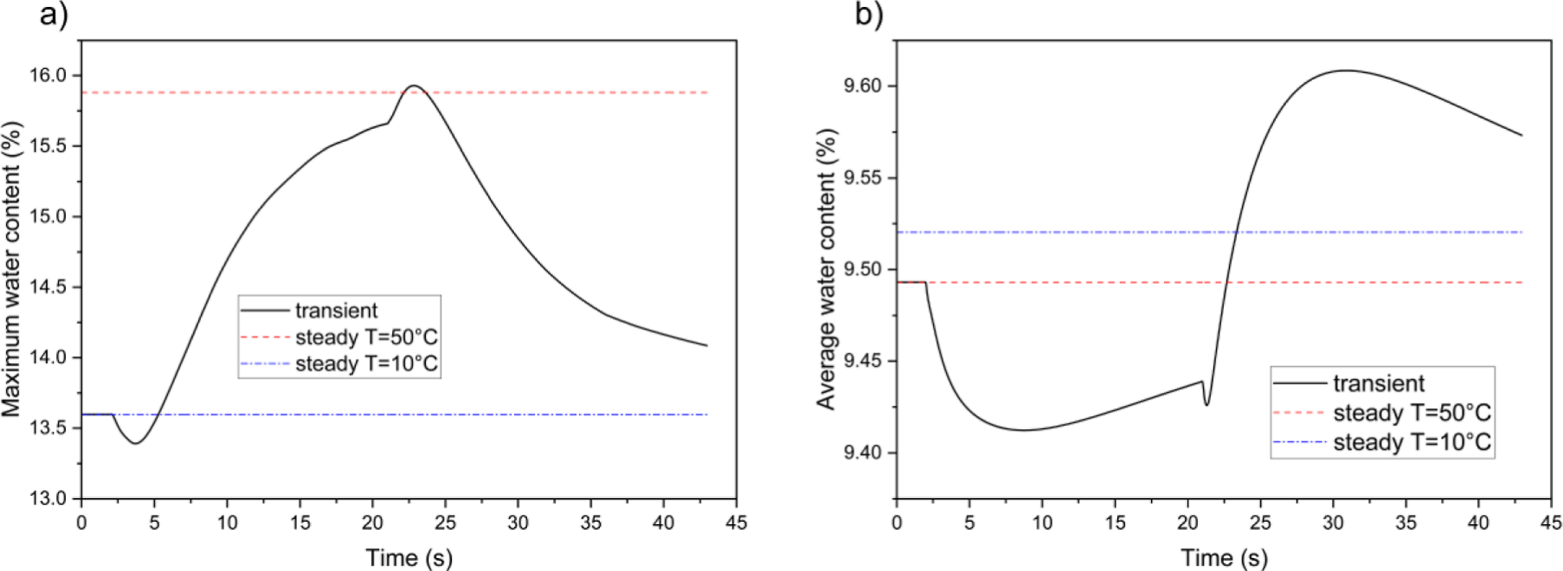
Local water content in the membrane for the case with a long-period thermal cycle; (a) maximum values, (b) average values.

The water content of the membrane is a crucial parameter that significantly influences the performance and efficiency of the fuel cell. Figure 5 illustrates the variations in both the maximum and average local values of water content within the membrane during the simulation. As shown in Figure 5a, the steady-state value of maximum water content at cold temperatures is notably higher than at normal temperatures. Initially, the maximum water content deviates from the steady-state value in response to temperature changes, followed by a rapid adjustment back toward the steady state, although the rate of change gradually decreases. This slower rate of change leads to a prolonged process of reaching the steady-state level. During the cold conditions, the maximum water content first declines but then begins to increase, although it does not reach the steady-state value by the end of the cold period due to the reduced rate of increase. Once the temperature returns to normal, the maximum water content undergoes rapid fluctuations before slowly approaching the steady-state value. The maximum water content is concentrated in a high-water content layer within the membrane, typically closer to the cathode side. At lower temperatures, this high-water content layer becomes thinner, while the gradients of water content from the surface to the core increase. This behavior may be attributed to the fact that in colder conditions, both the rate of mass exchange between liquid water and vapor and the diffusion coefficient of water content are reduced. Further research may be needed to clarify these observations.

The response of the average water content to rapid temperature changes, as shown in Figure 5b, highlights how sudden shifts in temperature can lead to significant departures from equilibrium conditions. Similar to the maximum water content, the average water content reacts in the opposite direction from the new steady-state values after a temperature change. When the temperature drops, the average water content quickly declines, moving away from the new steady-state value. Once the temperature returns to normal, the average water content rapidly increases, overshooting the steady-state value before slowly declining. Even as it decreases, the curve remains above the steady-state value for the remainder of the simulation.

Simulations with a shorter thermal cycle show that both the maximum and average water content in the membrane deviate from their normal values, and with a slow return to their steady-state levels, they remain significantly higher than normal throughout the entire simulation. The slight increase in current density after *t* > 25 s aligns with observations in Figures S11 and 5, where the minimum oxygen concentration and membrane water content (both maximum and average) surpass the steady-state values. To better understand which of these parameters is most influential, an additional simulation was conducted with the same conditions, except that the oxygen stoichiometric ratio was set to λ_O_2__ = 1.5. The results for *i* and the minimum *f*_O_2__ (Figure S13) demonstrate that despite the minimum falling below the steady-state value after the shock, the current density remained higher than the steady-state value. This indicates that the elevated membrane water content is likely the main factor responsible for the fuel cell’s overperformance following the temperature cycle.

These results suggest that the minimum *f*_H_2__ at the anode CL, the minimum *f*_O_2__ at the cathode CL, and the average water content in the membrane are key parameters in the fuel cell’s dynamics. They provide critical information useful for predicting performance. Furthermore, liquid water saturation in the GDLs offers valuable insights into the behavior of the system.

### 3.3 Repeated Thermal Shocks

In this study, 19 thermal shocks are introduced as shown in Figure S14 (Appendix), each lasting for 1 s with a 1 s interval between them. During each cycle, the temperature of the bipolar plate (BP) walls is reduced to 10 °C. The total duration of the cold conditions in this simulation is equivalent to that of the cold conditions in Section 3.2, allowing for meaningful comparisons between the single-cycle and multicycle cases. This comparison will help to better understand how the duration and frequency of the cycles impact performance and key parameters.

**Figure 6:**
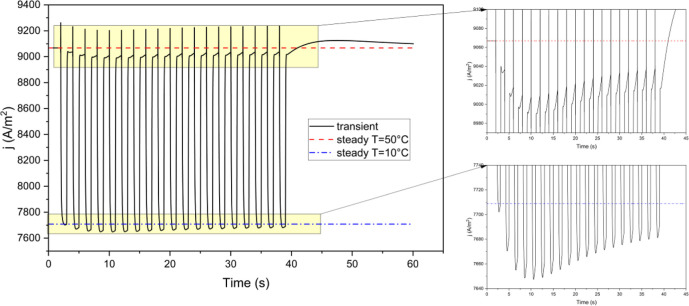
Current density during the case with repeated thermal cycles. Inset figures shows the specific ranges during the cycles.

Figure 6 illustrates the variations in current density over time. Under multiple short thermal cycles, the fuel cell performance is worse than in the single-cycle case, with the current density falling substantially below steady-state levels for nearly the entire duration of the cycles. During thermal cycling, the average current density, *j*, in the low-temperature conditions decreases cyclically, meaning its value at a given point in one cycle is smaller than at the same point in the previous cycle. This decline continues until cycle 5, after which the current density begins to increase. A similar pattern is observed during normal temperature conditions, where the current density decreases until cycle 5 and then starts to rise. In cycle 5, both at low and normal temperatures, the current density is approximately 0.7% lower than the steady-state value at the corresponding temperature. After the final cycle, the current density surpasses the steady-state value and remains above it for the remainder of the simulation.

**Figure 7:**
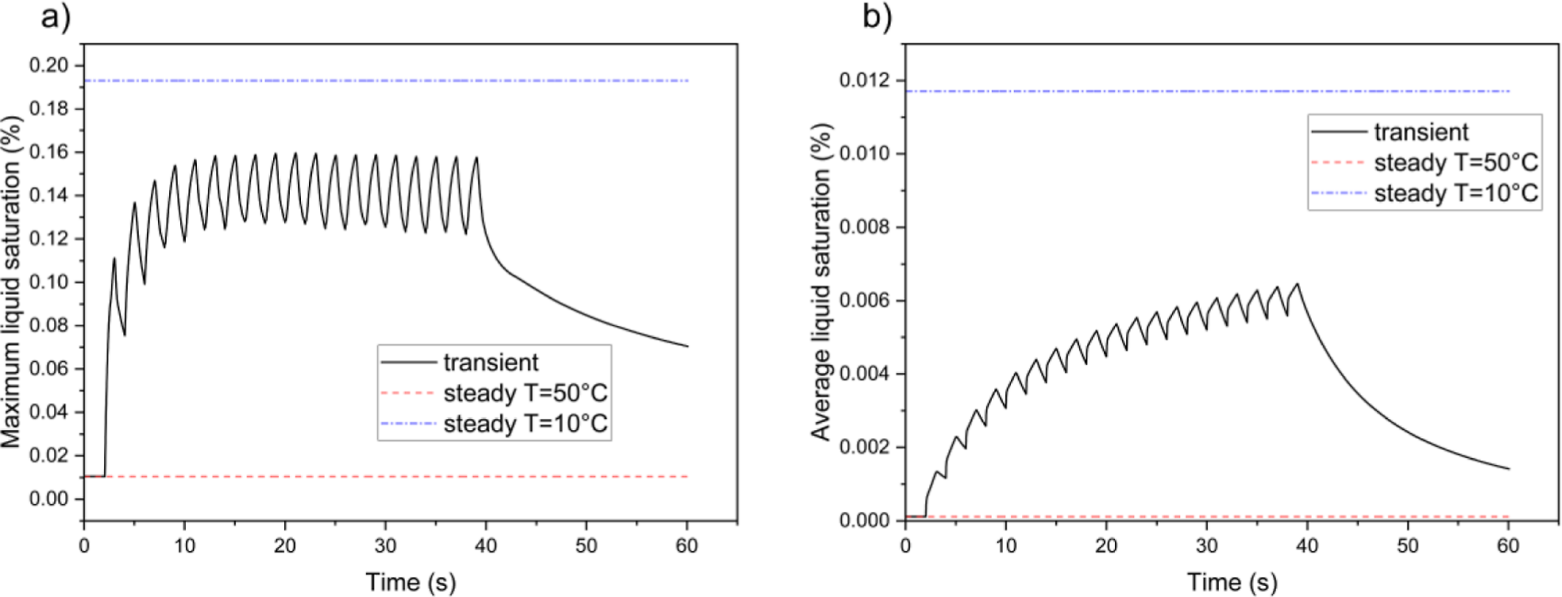
Variations of liquid water saturation (*s*) inside anode GDL in the case with repeated thermal cycles; (a) maximum water saturation vs time, (b) average water saturation values vs time.

Figure 7 present the water saturation (*s*) curves in the anode GDL. After an initial rapid increase, the maximum water saturation reaches a cyclic stability that remains significantly lower than the steady-state value. The curve for the average water saturation shows a continuous cyclic rise, with temperature-dependent oscillations occurring within each cycle. However, the peak of this curve remains noticeably lower than the steady-state value under cold conditions.

The saturation curves for the cathode side are presented in Figure S15 (Appendix). Overall, both the maximum and average saturations exhibit temperature-dependent fluctuations within each cycle. However, saturation levels tend to increase toward the end of each cycle, and this upward trend persists throughout the remaining cycles. Regarding the maximum saturation, the first cycle follows a different pattern than the subsequent cycles. Similar to the previous case with a single long cycle, the maximum saturation initially decreases during the cold conditions of the first cycle, followed by a rise. A comparison between the *s*–*T* curves of the first and last cycles reveals that the average saturation approaches cyclic stability by the last cycle. Most of the growth in maximum saturation occurs at the end of the cycles, coinciding with the return of the temperature to normal levels. Both curves begin to decline after the last cycle; however, the maximum saturation curve shows a slight delay before starting its descent.

When comparing the saturation curves between the single-cycle and multiple-cycle cases, and disregarding the fluctuations, the variation patterns for both maximum and average values show significant similarities. However, in the multiple-cycle case, the peaks of the curves are generally smaller. On the anode side, the curve for maximum saturation reaches a stability close to the steady-state point. In the multiple-cycle scenario, the additional liquid water formed during cold conditions remains in the porous media for a relatively long period. Moreover, changes in the amount of liquid water occur primarily when the temperature field is stabilized, a trend that is more pronounced in the case of long-period temperature cycles.

The dynamics of minimum oxygen mole fraction *f*_O_2__, in relation to temperature throughout the simulation is illustrated in Figure S16 (appendix). As the temperature returns to normal, the curves for minimum, *f*_O_2__ fall below their respective paths during the temperature decrease phase. Interestingly, the curves for all cycles follow the same trajectory, including the case with a single 3 s cold cycle (Figure S16). The curve for the single cycle with 19 s of cold conditions is also displayed in the figure. Since the system has more time to approach steady-state conditions at the lower temperature during the longer single cycle, the minimum *f*_O_2__ curve shifts slightly upward, nearing equilibrium at 10 °C. Consequently, the minimum *f*_O_2__ depends almost entirely on the temperature and the direction of temperature change.

**Figure 8:**
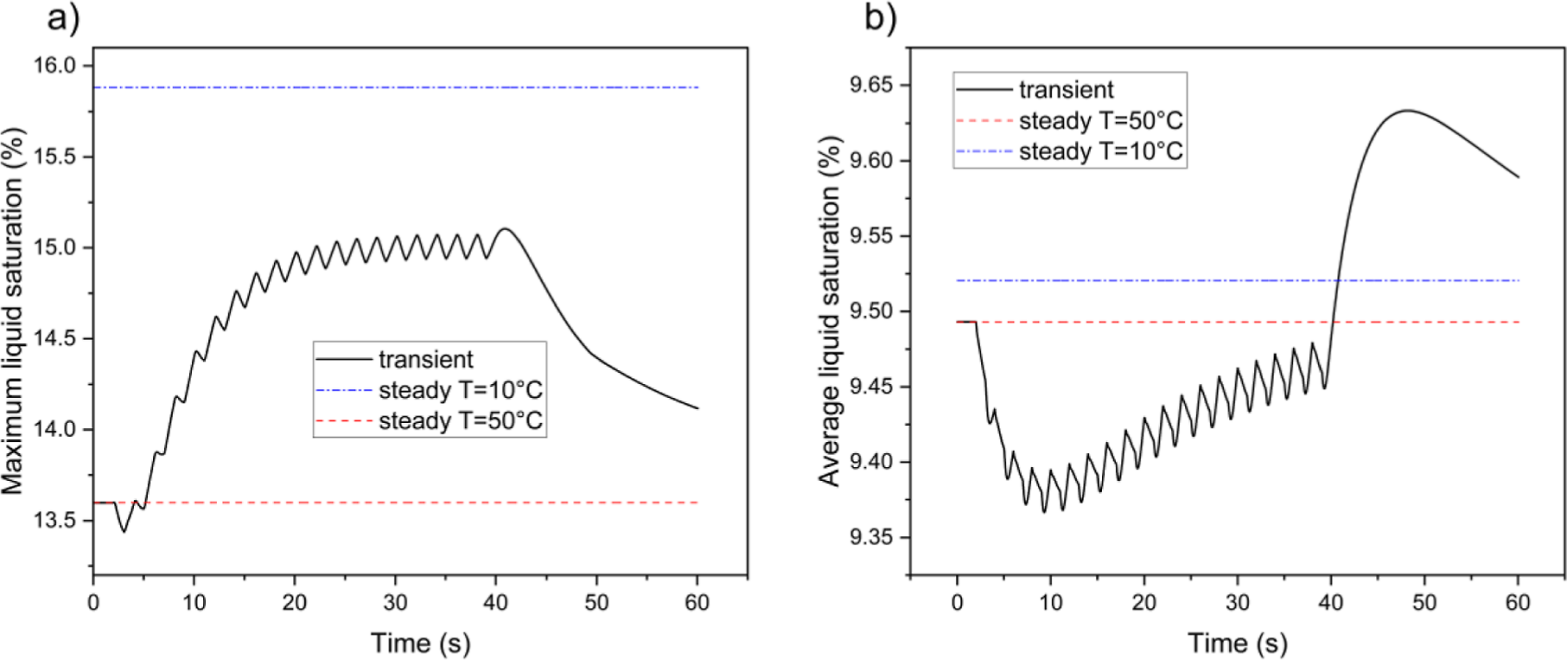
Local water content in the membrane for the case with repeated thermal cycles; (a) maximum values, (b) average values.

Figure 8 presents the curves for the maximum and average values of the local water content in the membrane. For the maximum value curve, aside from the first cycle, there is a cyclic increase where the maximum water content becomes progressively higher from one cycle to the next at similar points. Over time, the curve approaches cyclic stability, meaning that after a few cycles, the changes between cycles stabilize. The values at the end of each cycle become nearly identical to those of the previous cycle, indicating that the system has achieved steady oscillatory behavior.

The overall pattern of changes for both maximum and average values of the membrane water content is similar to that seen in the single-cycle case. However, with multiple cycles, the peak of the maximum water content is significantly lower than in the single long cycle, while the average values fluctuate within a wider range. This suggests that a long-period cycle tends to increase the gradients of water content within the membrane more than multiple short cycles. Even after cyclical stabilization, the maximum water content remains noticeably lower than the steady-state value under cold conditions.

The average water content during the cycles is also consistently lower than the steady-state value. However, after the initial rapid declines during the first few cycles, the average values begin to increase toward the steady-state level. Once the temperature cycles are completed, the average water content rises rapidly and stays above the steady-state level for the remainder of the simulation. The rate of return to equilibrium is significantly faster than in the single-cycle case. Cyclic variations in current density follow a similar pattern to the curve for the average membrane water content, suggesting that membrane water content plays a key role in influencing the fuel cell’s performance throughout the cycles. It is important to note that our findings are consistent with those reported in several studies referenced in the literature of this research.

## 4 Conclusions and Future Work

This study investigated the transient performance of a polymer electrolyte membrane fuel cell (PEMFC) in response to temperature variations, simulating conditions relevant to aerospace conditions. By applying thermal shocks to the bipolar plates’ walls, the study revealed key insights into the dynamic response of the fuel cells. While the terminal voltage remained fixed, the current density was calculated as the primary output. The main findings are as follows:

- Temperature reductions significantly decreased current density and reactant consumption, particularly near the stack edges and downstream sections, where oxygen concentrations were lowest. Cold conditions also led to excess liquid water accumulation near hydrogen inlets, obstructing reactant flow.
- The membrane’s water content exhibited transient deviations, with reduced water content during cooling and overshooting steady-state levels upon recovery. These variations, influenced by slow liquid water dynamics in porous media, emphasize the importance of managing water balance during normal cycles.
- Recovery patterns varied across cell parameters. After an initial decline in performance, the cell exhibited a temporary improvement in efficiency postrecovery, driven by transient changes in hydrogen, oxygen, and water content.

These findings provide practical insights for addressing transient thermal management, reactant distribution, and water management in PEMFCs for aviation. The parallel branched channel layout with counterflow, suitable for moderate-load conditions, was found to offer a balance between efficiency and simplicity. However, its performance under dynamic aerospace-specific scenarios requires further investigation. Future research should focus on:

- Experimental validation of numerical findings and optimization of PEMFC designs for aerospace applications.
- Exploring alternative flow channel configurations, such as serpentine and hybrid designs, to enhance performance under transient thermal and water management challenges.
- Integrated advanced cooling and humidification systems to improve reliability in extreme aerospace conditions.
- Scaling these findings to larger fuel cell stacks for high-power aviation applications.

This study highlights the potential of PEMFCs as efficient and sustainable energy systems for aviation. Collaborative efforts between researchers, industry, and policymakers will be essential to overcome technical challenges and realize their full potential in aerospace applications.

## Data Availability

No data is associated with this paper.

## Supporting Information Available

Details of simulation setup (methodology). Figures S1–S3 present schematic diagrams supporting the fuel cell, while Figures S4–S16 provide Supporting Information (PDF)

## Nomenclature

A: ampere (A)
BP: bipolar plate
CL: catalyst layer
GDL: gas diffusion layer
MEA: membrane electrolyte assembly
PEM: polymer electrolyte membrane
PEMFC: polymer electrolyte membrane fuel cell
V: voltage (Volt)
