# Supplementary material for: Thermal Transient Performance of PEM Fuel Cells in Aerospace Applications: A Numerical Study
Source: Energy Fuels. 2025 Apr 15;39(16):7876–89. doi: 10.1021/acs.energyfuels.4c04834 (PMC12035800; doi:10.1021/acs.energyfuels.4c04834)
Supplement: Supplementary file 1 — ef4c04834_si_001.pdf [file ef4c04834_si_001.pdf]

Supporting Information

Thermal Transient Performance of PEM Fuel Cells in Aerospace Applications: A Numerical Study

Mehdi Seddiq[1,2], Mohammad Alnajideen[1,*], Rukshan Navaratne[1]

[1] College of Physical Sciences and Engineering, Cardiff University, Cardiff CF24 3AA, UK
[2] City University of London, School of Science and Technology, 280 St John Street, London, EC1V 4PB, UK

* Corresponding author: AlnajideenMI@cardiff.ac.uk

Methodology: Simulation Setup

Generating high-quality hexahedral (Hexa) elements with ANSYS-meshing software for the fuel cell geometry can be challenging. The channel geometry creates imprints on the neighboring GDL interface, and the software lacks the functionality to sweep between the faces of the GDLs. Without special provisions, tetrahedral (Tetra) elements are generated for the GDLs. Simulations with the Tetra elements showed that a considerably larger number of elements is generated with the same mesh size. More importantly, the residuals are generally larger, convergence is more difficult, and extra caution is needed to ensure the results are valid. To accomplish this, the geometries for all regions are split into smaller parts according to the channels' imprints on the GDLs. These parts do not appear in the ANSYS solver but are used only under the Geometry and Meshing toolkit. Using a Sweeping method, a fully Hexa mesh is generated for the resulting parts separately. However, this process is excessively laborious, and it is recommended to use more suitable meshing software for fuel cell modeling. ANSYS Fluent software, combined with the PEMFC module, is utilized to simulate the two-phase, multi-species fluid flow, heat transfer, and charge transport within a three-dimensional domain. The discretization in ANSYS Fluent is based on the Finite Volume Method (FVM).

The discretized equations are implicit, and the solution is obtained iteratively. The SIMPLE algorithm is employed for pressure-velocity coupling. For spatial discretization, the Green-Gauss Cell-Based method is used for gradients, the Standard scheme for pressure, and the First Order Upwind method for other variables. Temporal discretization is performed using the First Order Implicit method. On unstructured meshes, solving the implicit discretized equations is complex and can be particularly time-consuming for fuel cell simulations. Convergence is challenging, especially in the initial iterations and under changing conditions. ANSYS Fluent utilizes Algebraic Multigrid (AMG) methods to enhance stabilization and accelerate convergence. In this scheme, correction equations are solved across multiple coarse grid levels, following specific cycles to navigate through the grid hierarchy. The F-Cycle type, combined with the Bi-Conjugate Gradient Stabilized (BCGSTAB) method for all governing equations except for momentum, has proven effective.

Selecting appropriate under-relaxation factors is crucial for achieving convergence in fuel cell simulations. The under-relaxation factors applied are: 0.6 for pressure, 0.6 for momentum, 0.75 for species, 0.6 for energy, and values of 1 or slightly lower for density, body forces, electric and protonic potentials, capillary pressure, water content, and liquid saturation in channels. Special attention is given to source terms in liquid water and dissolved phase transport equations, with under-relaxation factors of 0.6 and 0.7 for mass exchange between liquid and vapor and between liquid and dissolved phases, respectively. For the gas diffusion layer (GDL) liquid water removal source term, an under-relaxation factor of 0.75 is used. A time step of 1 ms is utilized for most transient simulations. In near-stable conditions where variable curves show minimal changes, larger time steps of up to 5 ms are considered. Based on values from previous studies [1, 2] and considering that temperature changes, which drive transience, propagate slowly and affect chemical reactions gradually, the chosen time steps are expected to be adequately small.

Fuel cell physics involves several coupled transport phenomena, some of which are indirect or not evident from the equations. Therefore, determining true convergence is more complex than in standard fluid dynamics simulations. Relying on residual curves can be misleading, as new patterns and significant changes in current/voltage may emerge after several hundred iterations.

For steady-state simulations, convergence is accepted if residual curves continuously decrease and the maximum concentrations of hydrogen, oxygen, and water, velocities perpendicular to the membrane plane, and temperature remain constant. Our simulations indicate that when any of the following quantities stabilize, all others do as well: maximum hydrogen mole fraction at the anode catalyst layer (CL)-GDL interface, minimum oxygen mole fraction at the cathode CL-GDL interface, maximum pressure at the anode outlet, and the average water content in the membrane. These quantities can be used as convergence criteria alongside residual curves. In our simulations, a pseudo-steady state is reached after 1500-2000 iterations, although key parameters may not fully stabilize until 6000 iterations. In transient runs initialized with a converged steady-state solution, minor fluctuations in output current density are observed for a fraction of a second. A fully converged steady-state solution is accepted after a 0.4-second transient run using the steady-state solution as the initial condition.

Supporting figures and data

The first three figures present supporting graphics, while the remaining figures provide supporting data.

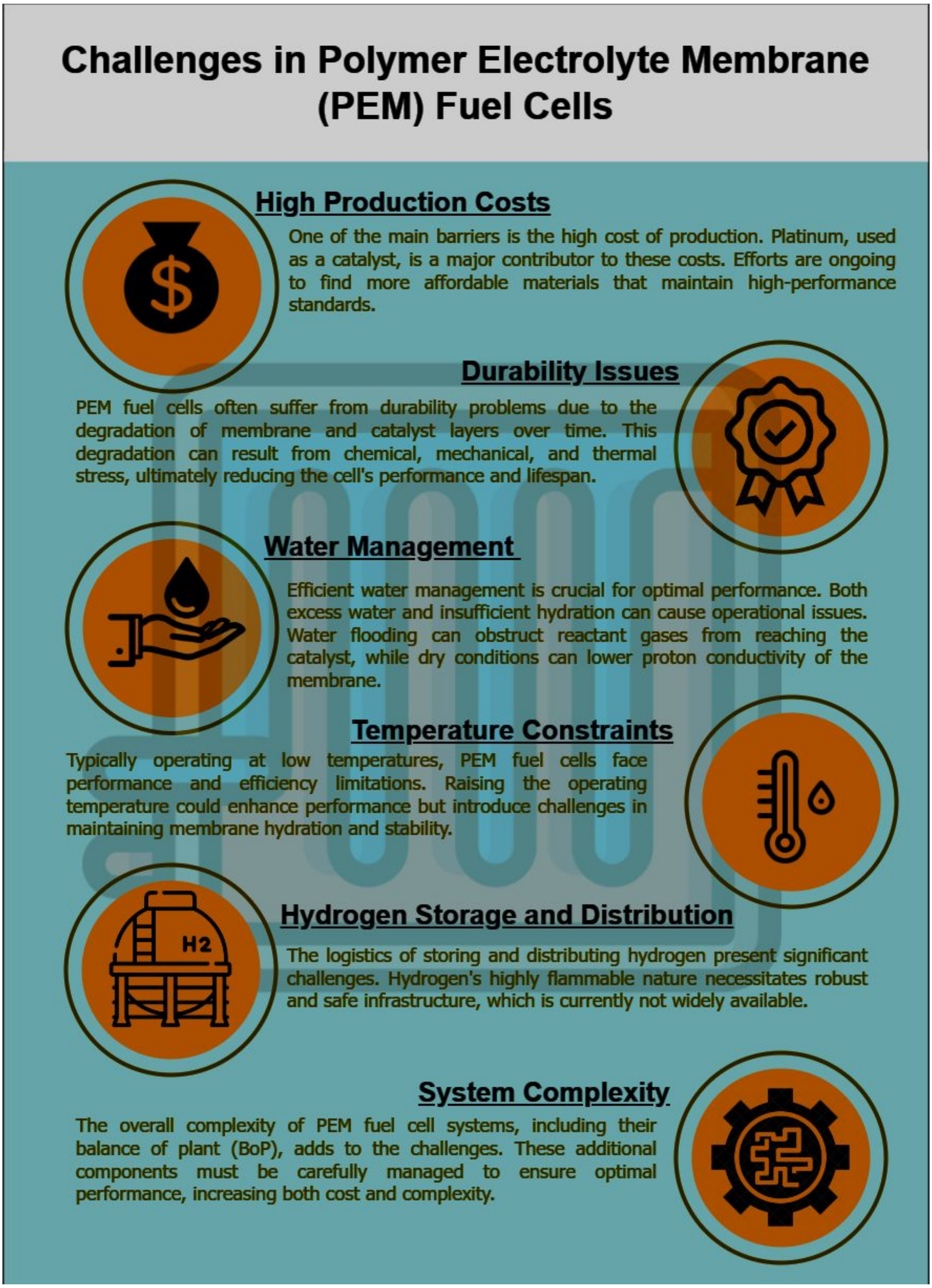

Fig. S1: Key primary barriers that face Polymer Electrolyte Membrane (PEM) fuel cells. Authors created the infographic.

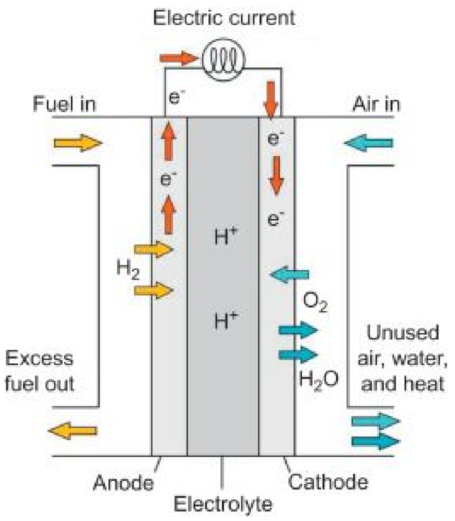

Fig. S2: The structure of a single PEM fuel cell. Reprinted with permission from ref ([3]), Copyright 2024 Elsevier.

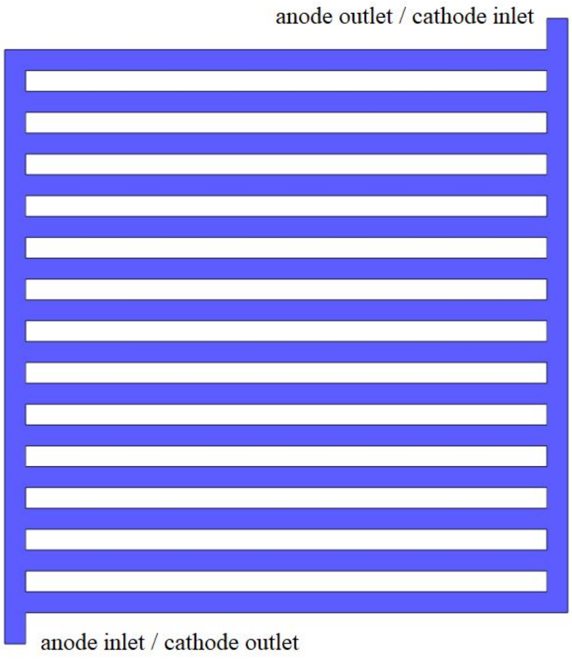

Fig. S3: Channel layout for the fuel cell model.

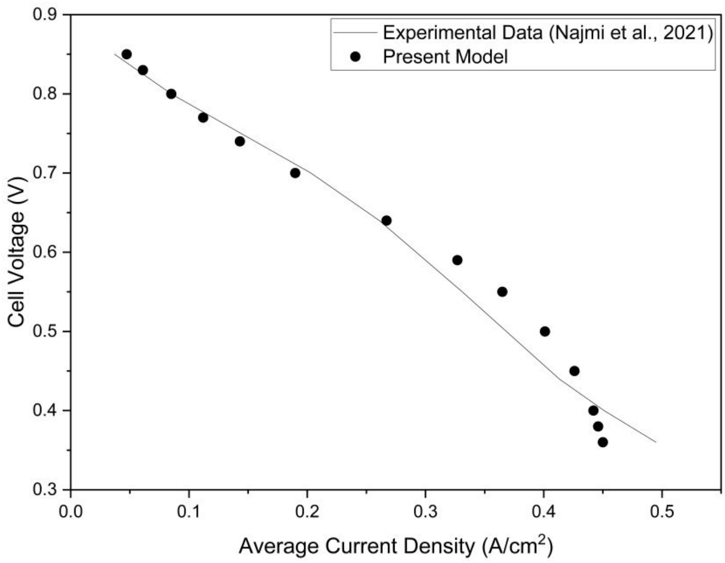

Fig. S4: Comparison of the model polarization curve with the experimental data from Najmi et al. [4].

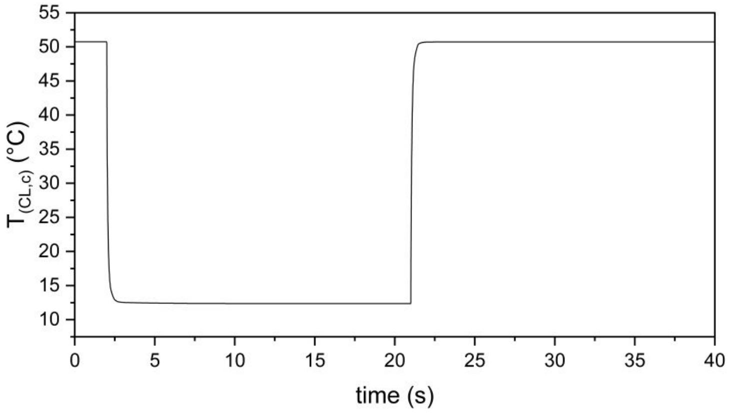

Fig. S5: Variations of the average temperature for the cathode catalyst ($T_{CL,c}$) during the case with a single short-period thermal cycle ($t = 40s$).

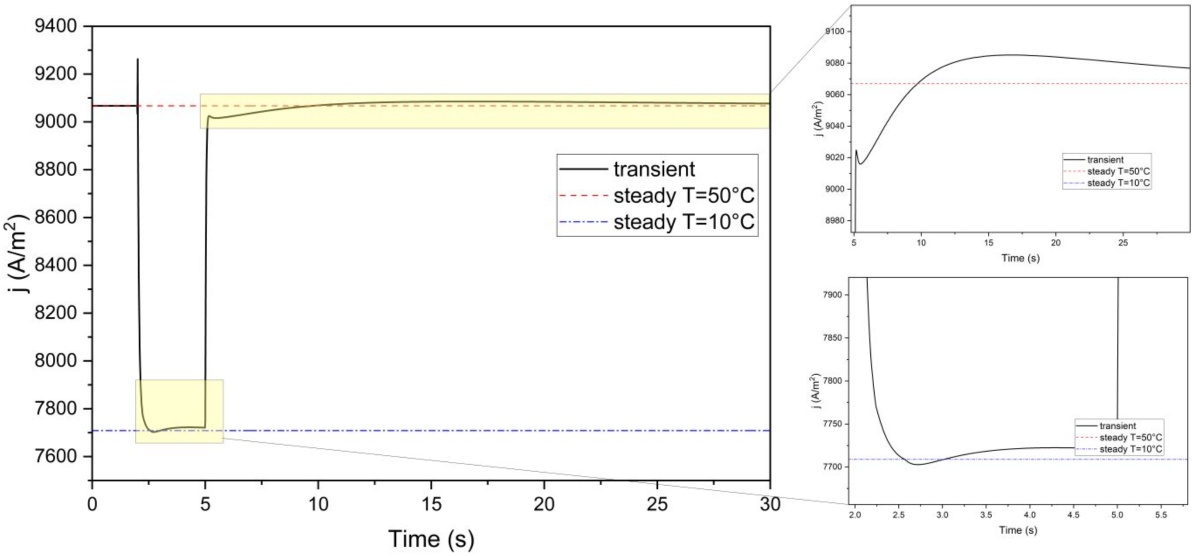

Fig. S6: Variations in current density during the transient simulation with a single short thermal cycle (inset figures show detailed views for specific time intervals at cold and warm steady-state conditions).

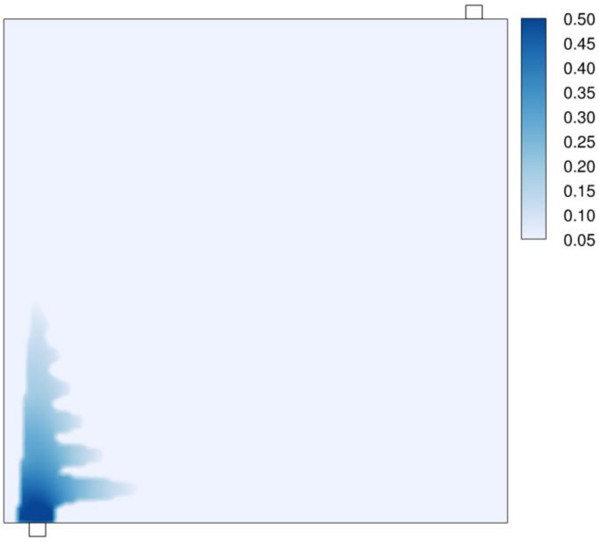

Fig. S7: Distribution of liquid water saturation at the anode CL-GDL interfaced during a long-period temperature cycle at $t = 20.5s$*. The rectangles in the bottom left and upper right show the inlet and outlet of the PEM cell, respectively.*

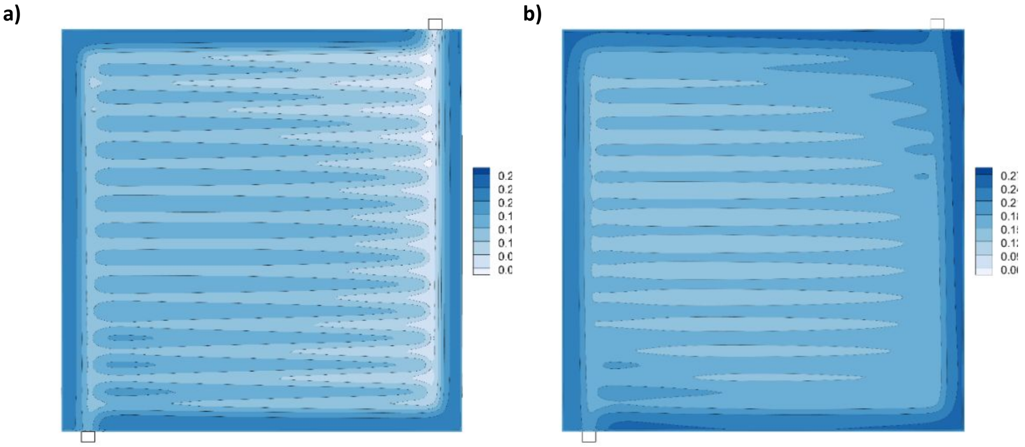

Fig. S8: Distribution of liquid water saturation at the interface of cathode CL-GDL for a long-period temperature cycle; a) at $t = 1.5s$ (steady-state condition at $T = 50°C$); and b) $t = 20.5s$ (clos to the end of cold-temperature conditions).

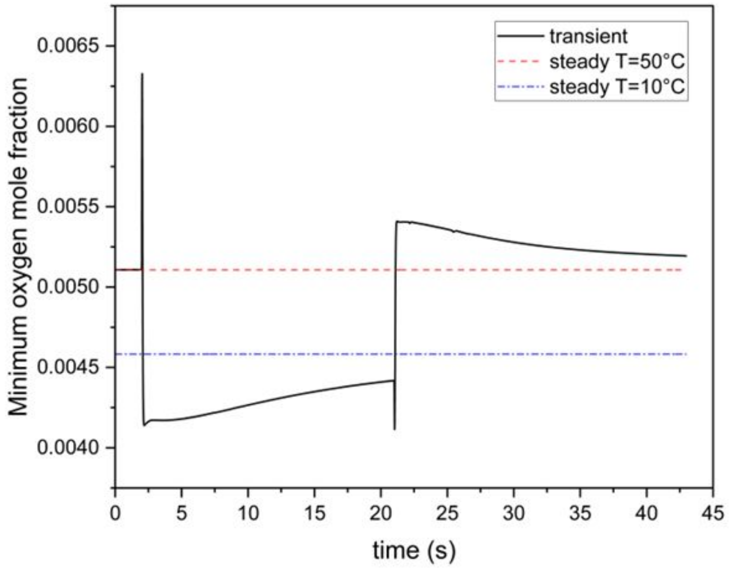

Fig. S9: Minimum values of oxygen mole fractions at cathode GDL-CL interface in the case with a long-period thermal cycle.

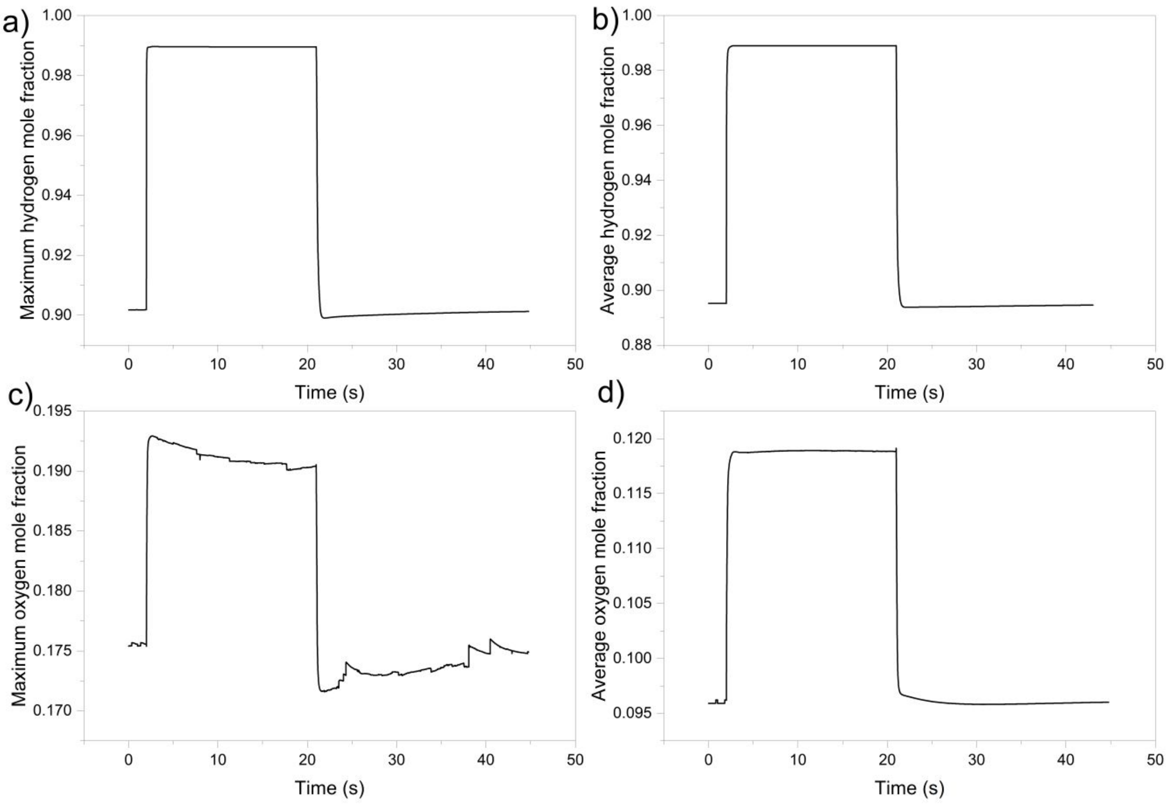

Fig. 10: a) Maximum values of hydrogen mole fractions at anode GDL-CL interface in the case with a long-period thermal cycle; b) Average values of hydrogen mole fractions at anode GDL-CL interface in the case with a long-period thermal cycle; c) Maximum values of oxygen mole fractions at cathode GDL-CL interface in the case with a long-period thermal cycle; and d) Average values of oxygen mole fractions at cathode GDL-CL interface in the case with a long-period thermal cycle.

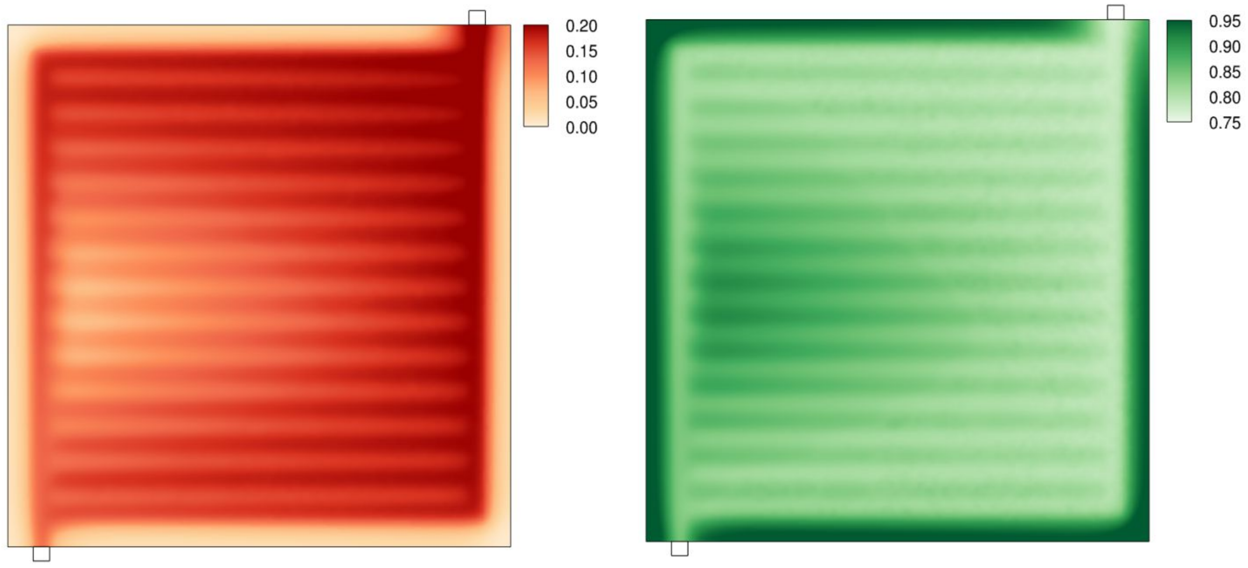

Fig. S11: Mole fraction distributions at cathode GDL-CL interface at t=20.5s (close to the end of cold-temperature conditions), a) Oxygen, b) Nitrogen. The rectangles in the upper right and bottom left are inlet and outlet, respectively.

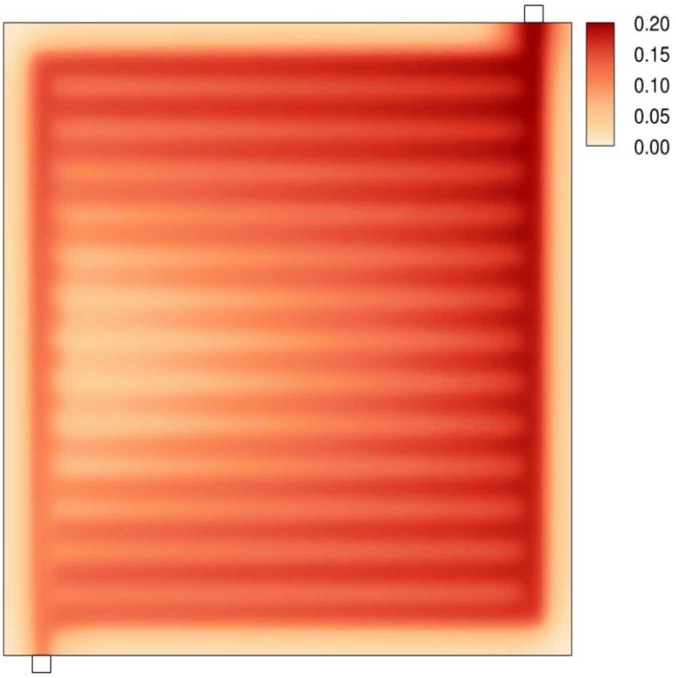

Fig. S12: Oxygen mole fraction distributions at cathode GDL-CL interface in steady state T=50°C. The rectangles in the upper right and bottom left are inlet and outlet, respectively.

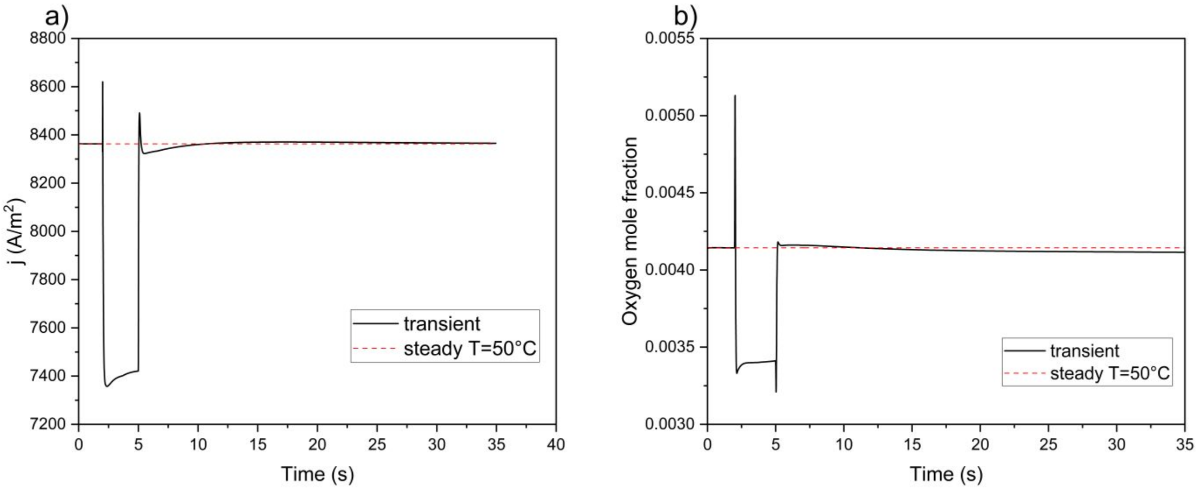

Fig. S13: a) Current density and minimum f_{O_2} at cathode GDL-CL interface in a simulation with a single thermal cycle and $\lambda_{O_2} = 1.5$.

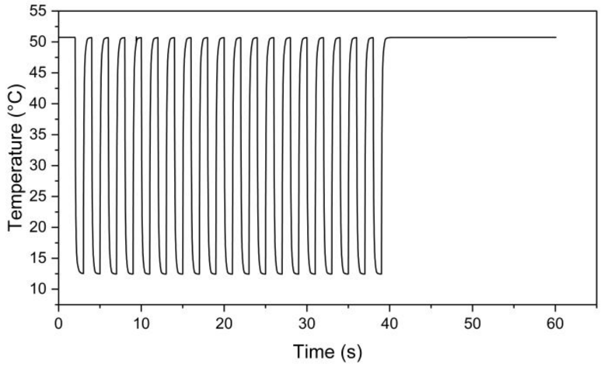

Fig. S14: The average temperature for the cathode catalyst, $T_{CL,c}$, during the case with repeated thermal cycles.

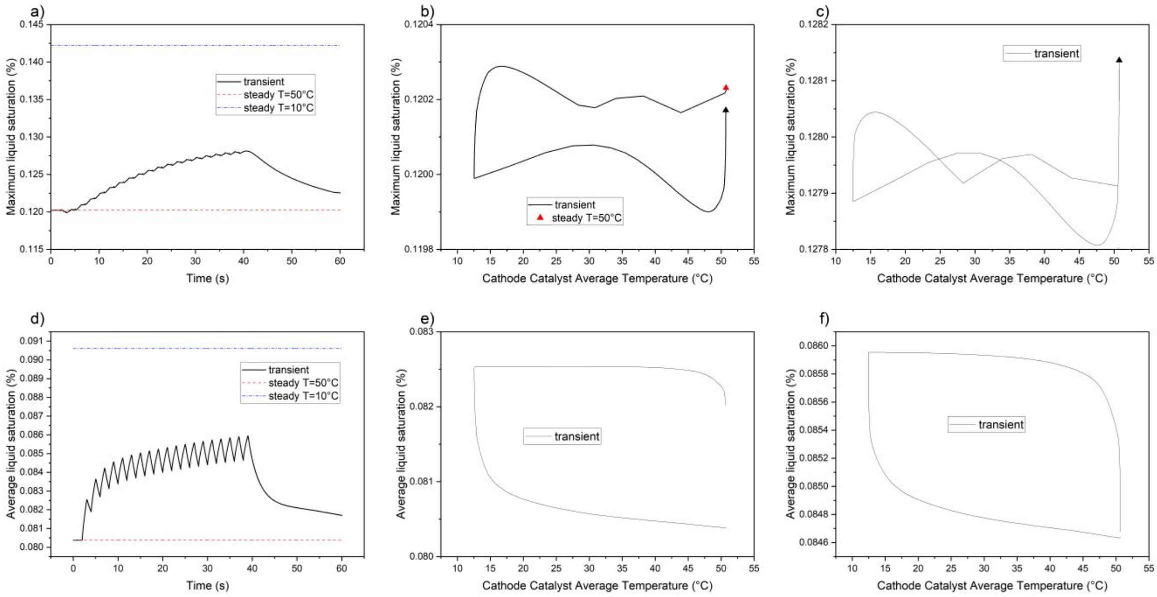

Fig. S15: Variations of liquid water saturation (s) inside cathode GDL in the case with repeated thermal cycles; a) maximum s vs time, b and c) maximum s vs temperature during the first and last (19th) cycles, d) average s values vs time, e and f) averages vs temperature during the first and last (19th) cycles.

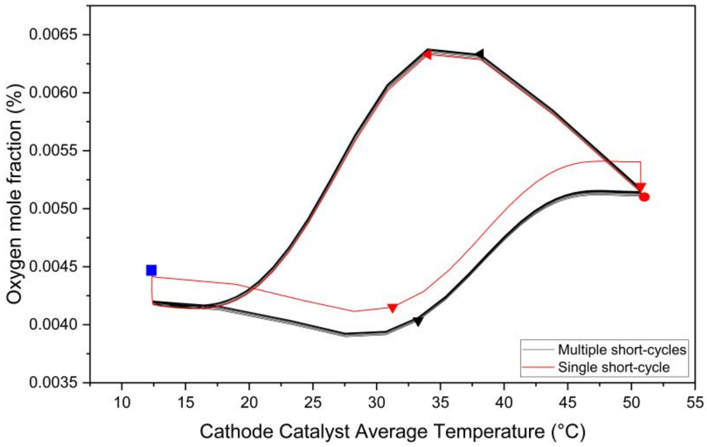

Fig. S16: Minimum mole fractions of oxygen at cathode GDL-CL interface vs temperature (T_{CL_c}) in the case with repeated thermal cycles. The path from high to low temperature is the upper curve.

References

[1]. Goshtasbi, A., et al., *Through-the-Membrane Transient Phenomena in PEM Fuel Cells: A Modeling Study.* Journal of The Electrochemical Society, 2019. **166**(7): p. F3154-F3179.

[2]. Bodner, M., et al., *Simulation-Assisted Determination of the Start-Up Time of a Polymer Electrolyte Fuel Cell.* Energies, 2021. **14**: p. 7929.

[3]. Breeze, P., *Chapter 4 - The Proton Exchange Membrane Fuel Cell*, in *Fuel Cells*, P. Breeze, Editor. 2017, Academic Press. p. 33-43.

[4]. Najmi, A.-U.-H., et al., *Experimental investigation and optimization of proton exchange membrane fuel cell using different flow fields.* Energy, 2021. **217**: p. 119313.
